# Redetermination of bis­{(1*S*,2*S*,4*S*,5*R*)-2-[(*R*)-hy­droxy(6-meth­oxy-4-quinol­yl)meth­yl]-5-vinyl­quinuclidinium} sulfate dihydrate

**DOI:** 10.1107/S1600536810034288

**Published:** 2010-08-28

**Authors:** Peter Mangwala Kimpende, Luc Van Meervelt

**Affiliations:** a Katholieke Universiteit Leuven, Department of Chemistry, Celestijnenlaan 200F, B-3001 Leuven (Heverlee), Belgium

## Abstract

The structure of the title compound, known as quinine sulfate dihydrate, 2C_20_H_25_N_2_O_2_^+^·SO_4_^2−^·2H_2_O, was previously reported by Mendel [*Proc. K. Ned. Akad. Wet.* (1955), **58**, 132–134], but only the [010] projection was determined. Hence, we have redetermined its crystal structure at 100 K using three-dimensional data. The asymmetric unit consists of a quininium cation, *viz.* (*R*)-(6-meth­oxy­quinolinium-4-yl)[(1*S*,2*S*,4*S*,5*R*)-5-vinyl­quinuclid­in­ium-2-yl]methanol, one half of a sulfate anion and a water mol­ecule. The S atom occupies a special position on a twofold axis. The packing is characterized by infinite columns, consisting of sulfate anions and water mol­ecules, linked through hydrogen bonds along the *b* axis, and further stabilized by hydrogen bonds to quininium cations. The quininium cations inter­act further through C—H⋯O and C—H⋯π inter­actions.

## Related literature

For the biological activity of quinoline-based anti­malarial drugs, see: Chou *et al.* (1980 ▶). For related structures and a previous determination of the title compound, see: Mendel (1955 ▶).
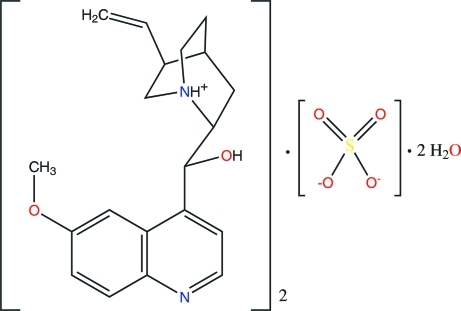


## Experimental

### 

#### Crystal data

2C_20_H_25_N_2_O_2_^+^·SO_4_^2−^·2H_2_O
*M* *_r_* = 782.94
Monoclinic, 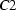

*a* = 20.180 (7) Å
*b* = 6.637 (2) Å
*c* = 15.316 (6) Å
β = 113.288 (9)°
*V* = 1884.2 (11) Å^3^
*Z* = 2
Cu *K*α radiation
μ = 1.31 mm^−1^
*T* = 100 K
0.24 × 0.15 × 0.04 mm

#### Data collection

Bruker SMART 6000 diffractometer
Absorption correction: multi-scan (*SADABS*; Bruker, 2003 ▶) *T*_min_ = 0.743, *T*_max_ = 0.949
9588 measured reflections
3319 independent reflections
3112 reflections with *I* > 2σ(*I*)
*R*_int_ = 0.063

#### Refinement

*R*[*F* ^2^ > 2σ(*F* ^2^)] = 0.039
*wR*(*F* ^2^) = 0.095
*S* = 1.04
3319 reflections
251 parameters
1 restraint
H-atom parameters constrained
Δρ_max_ = 0.32 e Å^−3^
Δρ_min_ = −0.29 e Å^−3^
Absolute structure: Flack (1983 ▶), 1339 Friedel pairs
Flack parameter: 0.00 (2)

### 

Data collection: *SMART* (Bruker, 2001 ▶); cell refinement: *SAINT* (Bruker, 2003 ▶); data reduction: *SAINT*; program(s) used to solve structure: *SHELXS97* (Sheldrick, 2008 ▶); program(s) used to refine structure: *SHELXL97* (Sheldrick, 2008 ▶); molecular graphics: *PLUTON* (Spek, 2009 ▶) and *DIAMOND* (Brandenburg, 2010 ▶); software used to prepare material for publication: *PLATON*.

## Supplementary Material

Crystal structure: contains datablocks I, global. DOI: 10.1107/S1600536810034288/lx2164sup1.cif

Structure factors: contains datablocks I. DOI: 10.1107/S1600536810034288/lx2164Isup2.hkl

Additional supplementary materials:  crystallographic information; 3D view; checkCIF report

## Figures and Tables

**Table 1:** Hydrogen-bond geometry (Å, °) *Cg*1 and *Cg*2 are the centroids of the C2–C10 and N1–C5 rings, respectively.

*D*—H⋯*A*	*D*—H	H⋯*A*	*D*⋯*A*	*D*—H⋯*A*
C11—H11⋯O4^i^	1.00	2.59	3.584 (3)	171
C16—H16*A*⋯O3	0.99	2.36	3.345 (3)	176
C17—H17*B*⋯O2^ii^	0.99	2.33	3.174 (3)	143
N2—H2*N*⋯O5	0.93	1.77	2.698 (3)	175
O2—H2⋯O3	0.84	1.87	2.695 (2)	166
O3—H3*A*⋯O5^iii^	0.86	1.99	2.765 (3)	149
O3—H3*B*⋯O4	0.86	1.97	2.794 (3)	159
C6—H6⋯*Cg*2^iv^	0.95	2.67	3.482 (3)	144
C20—H20*B*⋯*Cg*1^v^	0.95	2.85	3.530 (3)	130

Symmetry codes: (i) 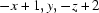; (ii) 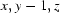; (iii) 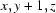; (iv) 
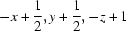
; (v) 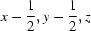.

**Fig. 1.**
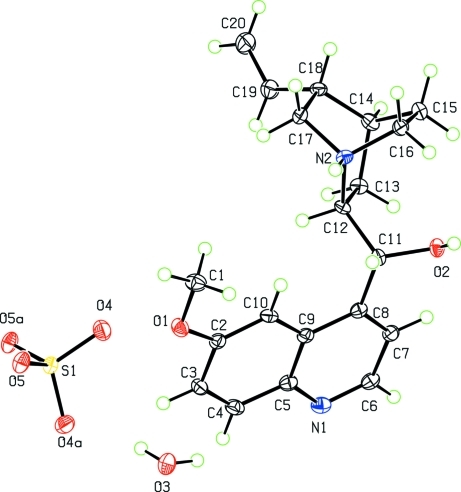
The molecular structure of the title compound with the atom numbering scheme. Displacement ellipsoids are drawn at the 50% probability level. H atoms are presented as small spheres of arbitrary radius.

**Fig. 2.**
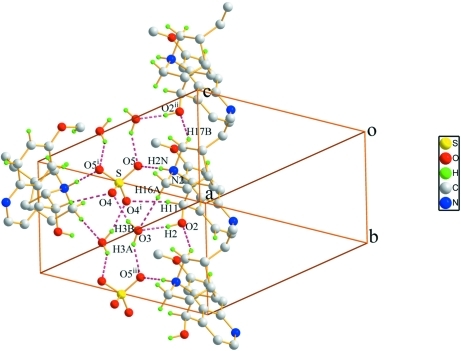
N—H···O and O—H···O interactions (dotted lines) in the crystal structure of the title compound. [Symmetry codes: (i) - x + 1, y, - z + 2; (ii) x, y - 1, z; (iii) x, y + 1, z]

**Fig. 3.**
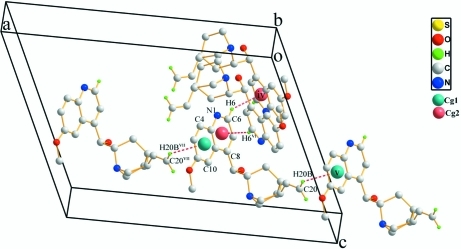
C—H···π interactions (dotted lines) in the crystal structure of the title compound. Cg denotes the ring centroid. [Symmetry codes: (iv) -x + 1/2, y + 1/2, -z + 1; (v) x - 1/2, y - 1/2, z; (vi) -x + 1/2, y - 1/2, -z + 1; (vii) x + 1/2, y + 1/2, z]

## supplementary crystallographic information

### Comment

Malaria is the most widespread and deadly humain infectious disease that is presently endemic in tropical and subtropical countries, covering approximately half of the world population. Its treatement is sometimes complicated with the appearence of antimalarial-resistant *Plasmodium falciparum* strains, arising in regions due to a long time use of a specific antimalarial drug molecule. Quinine, a quinoline core alkaloid extracted from the bark of cinchona tree, is considered in certain countries as medication of last resort against malaria and it is solely used to fight parasite strains that had resisted to other available antimalarial drug molecules.

According to Chou *et al.* (1980) the biological activity of quinoline-based antimalarial drugs is based on the formation of cytotoxic complexes between the latter molecules and ferriprotoporphyrin IX (hematin or hemin). The knowledge of the three-dimensional structure of such complexes should be a significant step towards the elucidation of its mechanism of action and the design of new antimalarial drugs. In an attempt to crystallize porphyrin-quinine complexes, quinine sulfate dihyrate was cocrystallized with the acidic form of [Fe(III) *meso*-tetra(4-sulfonatophenyl)porphine]chloride at pH 4.8 from a water/propyleneglycol mixture. However, the title compound was obtained of which the [010] projection of the crystal structure has previously been determined (Mendel, 1955). Hence, we have redetermined the structure at 100 K (Fig. 1).

The unit cell comprises two formula units; each of them consists of one sulfate anion, two water molecules, and two quininium cations, (*R*)-(6-methoxyquinolinium-4-yl)[(1*S*,2*S*,4*S*,5*R*)-5-vinylquinuclidinium-2-yl]methanol. The sulfur atom occupies a special position on a twofold axis; both cations are related by a twofold axis. The quinoline ring is planar; the maximal deviation (0.026 (2) Å) from the best plane is observed for C10. The angle between the best planes through the quinoline rings of both cations is 58.8 (1)°. The packing is characterized by infinite columns along the *b*-axis, in which sulfate anions and water molecules are alternately tied together through hydrogen bonds O3—H3B···04 and O3—H3A···O5 (Fig. 2, Table 1). These columns interact further with the quininium cations by hydrogen bonding (N2—H2N···O5, O2—H2···O3, C16—H16A···O3, C11—H11···O4; Table 1). The quininium cations interact further with each other by C—H···π (Fig. 3, Table 1) interactions and a C—H···O interaction (C17—H17B···O24; Table 1).

### Experimental

Quinine sulfate dihydrate, purchased from Acros Organics (Geel, Belgium), was added to the acid form of [Fe(III)*meso*-tetra(4-sulfonatophenyl)porphine]chloride (FeTSPP) in the mixture of water and propyleneglycol 6:4 to induce reaction between the two compounds at room temperature. The pH was fixed at 4.8 using 0.01 *M* acetate buffer and adjusted with either HCl or NaOH. Colourless plate-like crystals, suitable for X-ray diffraction, were obtained within five to six h.

### Refinement

Hydrogen atoms attached to N2 and O3 were located in a difference Fourier map. The other hydrogen atoms were positioned with idealized geometry using a riding model with C—H = 0.95–0.99 Å. All hydrogen atoms were further refined with isotropic temperature factors fixed at 1.2 or 1.5 times *U*_eq_ of the parent atoms.

### Crystal data

**Table:**

2C_20_H_25_N_2_O_2_^+^·SO_4_^2^^−^·2H_2_O	*F*(000) = 836
*M**_r_* = 782.94	*D*_x_ = 1.380 Mg m^−^^3^
Monoclinic, *C*2	Cu *K*α radiation, λ = 1.54178 Å
Hall symbol: C 2y	Cell parameters from 3041 reflections
*a* = 20.180 (7) Å	θ = 3.1–70.8°
*b* = 6.637 (2) Å	µ = 1.31 mm^−^^1^
*c* = 15.316 (6) Å	*T* = 100 K
β = 113.288 (9)°	Plate, colourless
*V* = 1884.2 (11) Å^3^	0.24 × 0.15 × 0.04 mm
*Z* = 2	

### Data collection

**Table:**

Bruker SMART 6000 diffractometer	3319 independent reflections
Radiation source: fine-focus sealed tube	3112 reflections with *I* > 2σ(*I*)
crossed Globel mirrors	*R*_int_ = 0.063
ω and φ scan	θ_max_ = 70.7°, θ_min_ = 3.1°
Absorption correction: multi-scan (*SADABS*; Bruker, 2003)	*h* = −24→23
*T*_min_ = 0.743, *T*_max_ = 0.949	*k* = −8→7
9588 measured reflections	*l* = −18→18

### Refinement

**Table:**

Refinement on *F*^2^	Secondary atom site location: difference Fourier map
Least-squares matrix: full	Hydrogen site location: difference Fourier map
*R*[*F*^2^ > 2σ(*F*^2^)] = 0.039	H-atom parameters constrained
*wR*(*F*^2^) = 0.095	*w* = 1/[σ^2^(*F*_o_^2^) + (0.0484*P*)^2^] where *P* = (*F*_o_^2^ + 2*F*_c_^2^)/3
*S* = 1.04	(Δ/σ)_max_ < 0.001
3319 reflections	Δρ_max_ = 0.32 e Å^−^^3^
251 parameters	Δρ_min_ = −0.29 e Å^−^^3^
1 restraint	Absolute structure: Flack (1983), 1339 Friedel pairs
Primary atom site location: structure-invariant direct methods	Flack parameter: 0.00 (2)

### Special details

**Table:**

Geometry. All esds (except the esd in the dihedral angle between two l.s. planes) are estimated using the full covariance matrix. The cell esds are taken into account individually in the estimation of esds in distances, angles and torsion angles; correlations between esds in cell parameters are only used when they are defined by crystal symmetry. An approximate (isotropic) treatment of cell esds is used for estimating esds involving l.s. planes.
Refinement. Refinement of *F*^2^ against ALL reflections. The weighted *R*-factor *wR* and goodness of fit *S* are based on *F*^2^, conventional *R*-factors *R* are based on *F*, with *F* set to zero for negative *F*^2^. The threshold expression of *F*^2^ > σ(*F*^2^) is used only for calculating *R*-factors(gt) *etc*. and is not relevant to the choice of reflections for refinement. *R*-factors based on *F*^2^ are statistically about twice as large as those based on *F*, and *R*- factors based on ALL data will be even larger.

### Fractional atomic coordinates and isotropic or equivalent isotropic displacement parameters (Å^2^)

**Table:**

	*x*	*y*	*z*	*U*_iso_*/*U*_eq_	
S1	0.5000	0.51309 (11)	1.0000	0.01395 (17)	
O1	0.47279 (8)	0.1096 (3)	0.69000 (11)	0.0189 (3)	
O2	0.32201 (8)	0.9132 (3)	0.79914 (10)	0.0157 (3)	
H2	0.3533	0.9443	0.8528	0.024*	
O3	0.40574 (8)	0.9948 (3)	0.98222 (10)	0.0203 (3)	
H3A	0.4308	1.1028	0.9875	0.024*	
H3B	0.4378	0.9042	1.0097	0.024*	
O4	0.47838 (9)	0.6396 (3)	1.06305 (11)	0.0224 (4)	
O5	0.43932 (8)	0.3783 (3)	0.94218 (11)	0.0189 (4)	
C1	0.48547 (12)	0.0841 (4)	0.78784 (16)	0.0209 (5)	
H1A	0.5164	0.1934	0.8252	0.031*	
H1B	0.5094	−0.0456	0.8104	0.031*	
H1C	0.4394	0.0869	0.7951	0.031*	
C2	0.43495 (11)	0.2754 (4)	0.64592 (15)	0.0147 (4)	
C3	0.42548 (11)	0.2932 (4)	0.54931 (15)	0.0178 (5)	
H3	0.4423	0.1899	0.5203	0.021*	
C4	0.39215 (11)	0.4589 (4)	0.49829 (15)	0.0185 (5)	
H4	0.3874	0.4712	0.4343	0.022*	
C5	0.36455 (11)	0.6127 (4)	0.53810 (15)	0.0163 (4)	
C6	0.30432 (11)	0.9131 (4)	0.52007 (15)	0.0167 (4)	
H6	0.2819	1.0268	0.4823	0.020*	
C7	0.30701 (11)	0.9037 (4)	0.61389 (15)	0.0156 (4)	
H7	0.2859	1.0076	0.6370	0.019*	
C8	0.34015 (11)	0.7442 (4)	0.67093 (14)	0.0141 (4)	
C9	0.37165 (10)	0.5906 (4)	0.63416 (15)	0.0141 (4)	
C10	0.40826 (10)	0.4200 (4)	0.68725 (14)	0.0145 (4)	
H10	0.4143	0.4060	0.7517	0.017*	
C11	0.34041 (11)	0.7262 (4)	0.77006 (14)	0.0138 (4)	
H11	0.3896	0.6852	0.8157	0.017*	
C12	0.28570 (10)	0.5609 (3)	0.76677 (14)	0.0132 (4)	
H12	0.2928	0.4457	0.7293	0.016*	
C13	0.20590 (10)	0.6274 (4)	0.71813 (14)	0.0150 (4)	
H13A	0.2034	0.7728	0.7028	0.018*	
H13B	0.1818	0.5519	0.6581	0.018*	
C14	0.16724 (11)	0.5862 (4)	0.78526 (15)	0.0161 (5)	
H14	0.1155	0.6282	0.7539	0.019*	
C15	0.20490 (12)	0.7091 (4)	0.87635 (16)	0.0181 (5)	
H15A	0.1779	0.6971	0.9178	0.022*	
H15B	0.2064	0.8530	0.8604	0.022*	
C16	0.28208 (11)	0.6285 (4)	0.92844 (14)	0.0166 (4)	
H16A	0.3169	0.7416	0.9450	0.020*	
H16B	0.2864	0.5605	0.9880	0.020*	
C17	0.25215 (11)	0.2979 (4)	0.85222 (15)	0.0159 (5)	
H17A	0.2651	0.2279	0.9138	0.019*	
H17B	0.2601	0.2038	0.8072	0.019*	
C18	0.17165 (11)	0.3630 (4)	0.81314 (15)	0.0154 (5)	
H18	0.1538	0.3483	0.8652	0.018*	
C19	0.12712 (12)	0.2289 (4)	0.73183 (16)	0.0216 (5)	
H19	0.1365	0.2326	0.6757	0.026*	
C20	0.07601 (13)	0.1066 (4)	0.73313 (19)	0.0281 (6)	
H20A	0.0652	0.0991	0.7881	0.034*	
H20B	0.0500	0.0260	0.6791	0.034*	
N1	0.33113 (10)	0.7734 (3)	0.48223 (13)	0.0185 (4)	
N2	0.29863 (9)	0.4822 (3)	0.86512 (12)	0.0134 (4)	
H2N	0.3467	0.4438	0.8948	0.016*	

### Atomic displacement parameters (Å^2^)

**Table:**

	*U*^11^	*U*^22^	*U*^33^	*U*^12^	*U*^13^	*U*^23^
S1	0.0136 (3)	0.0123 (4)	0.0143 (3)	0.000	0.0038 (3)	0.000
O1	0.0200 (7)	0.0183 (9)	0.0194 (7)	0.0051 (6)	0.0089 (6)	0.0009 (7)
O2	0.0184 (7)	0.0137 (8)	0.0128 (6)	0.0003 (6)	0.0039 (6)	−0.0020 (6)
O3	0.0190 (7)	0.0168 (8)	0.0208 (7)	0.0013 (7)	0.0032 (6)	0.0005 (7)
O4	0.0266 (8)	0.0199 (9)	0.0218 (8)	0.0033 (7)	0.0108 (7)	−0.0009 (7)
O5	0.0150 (8)	0.0146 (9)	0.0226 (8)	0.0019 (6)	0.0024 (6)	0.0014 (7)
C1	0.0204 (10)	0.0243 (13)	0.0195 (10)	0.0045 (9)	0.0094 (9)	0.0054 (10)
C2	0.0112 (9)	0.0147 (12)	0.0187 (10)	−0.0007 (8)	0.0064 (8)	0.0003 (9)
C3	0.0189 (10)	0.0187 (12)	0.0196 (10)	−0.0018 (9)	0.0114 (9)	−0.0040 (10)
C4	0.0195 (10)	0.0236 (14)	0.0143 (9)	−0.0032 (9)	0.0087 (8)	−0.0021 (9)
C5	0.0144 (9)	0.0180 (12)	0.0160 (10)	−0.0019 (9)	0.0055 (8)	0.0009 (10)
C6	0.0158 (10)	0.0165 (11)	0.0161 (10)	−0.0013 (9)	0.0044 (8)	0.0032 (9)
C7	0.0154 (9)	0.0144 (11)	0.0175 (10)	−0.0004 (9)	0.0070 (8)	0.0010 (9)
C8	0.0105 (9)	0.0158 (11)	0.0143 (9)	−0.0032 (8)	0.0030 (7)	−0.0004 (9)
C9	0.0107 (9)	0.0167 (11)	0.0146 (9)	−0.0045 (8)	0.0047 (8)	−0.0022 (9)
C10	0.0128 (9)	0.0194 (12)	0.0128 (9)	−0.0024 (9)	0.0066 (8)	−0.0001 (9)
C11	0.0140 (10)	0.0133 (11)	0.0142 (9)	0.0017 (8)	0.0056 (8)	−0.0015 (9)
C12	0.0142 (9)	0.0167 (12)	0.0100 (9)	0.0004 (8)	0.0063 (7)	−0.0005 (8)
C13	0.0131 (9)	0.0182 (12)	0.0128 (9)	−0.0002 (8)	0.0042 (8)	0.0009 (9)
C14	0.0117 (9)	0.0210 (13)	0.0149 (9)	0.0028 (9)	0.0046 (8)	−0.0004 (9)
C15	0.0211 (11)	0.0161 (12)	0.0199 (10)	0.0006 (9)	0.0110 (9)	−0.0025 (10)
C16	0.0209 (10)	0.0161 (12)	0.0130 (9)	−0.0034 (9)	0.0069 (8)	−0.0024 (9)
C17	0.0195 (10)	0.0114 (11)	0.0175 (10)	−0.0008 (9)	0.0082 (8)	0.0005 (9)
C18	0.0151 (10)	0.0180 (12)	0.0157 (10)	−0.0041 (8)	0.0090 (8)	−0.0018 (9)
C19	0.0220 (11)	0.0215 (13)	0.0221 (11)	−0.0040 (10)	0.0096 (9)	−0.0046 (10)
C20	0.0257 (12)	0.0260 (15)	0.0348 (13)	−0.0075 (11)	0.0144 (10)	−0.0085 (12)
N1	0.0176 (8)	0.0223 (11)	0.0157 (8)	−0.0016 (8)	0.0066 (7)	0.0018 (8)
N2	0.0114 (7)	0.0144 (10)	0.0144 (8)	0.0000 (7)	0.0050 (6)	0.0027 (8)

### Geometric parameters (Å, °)

**Table:**

S1—O4	1.4703 (17)	C10—H10	0.9500
S1—O4^i^	1.4703 (17)	C11—C12	1.543 (3)
S1—O5^i^	1.4923 (16)	C11—H11	1.0000
S1—O5	1.4923 (16)	C12—N2	1.517 (3)
O1—C2	1.357 (3)	C12—C13	1.547 (3)
O1—C1	1.427 (3)	C12—H12	1.0000
O2—C11	1.417 (3)	C13—C14	1.541 (3)
O2—H2	0.8400	C13—H13A	0.9900
O3—H3A	0.8626	C13—H13B	0.9900
O3—H3B	0.8622	C14—C15	1.533 (3)
C1—H1A	0.9800	C14—C18	1.535 (4)
C1—H1B	0.9800	C14—H14	1.0000
C1—H1C	0.9800	C15—C16	1.538 (3)
C2—C10	1.372 (3)	C15—H15A	0.9900
C2—C3	1.420 (3)	C15—H15B	0.9900
C3—C4	1.362 (4)	C16—N2	1.500 (3)
C3—H3	0.9500	C16—H16A	0.9900
C4—C5	1.412 (3)	C16—H16B	0.9900
C4—H4	0.9500	C17—N2	1.506 (3)
C5—N1	1.367 (3)	C17—C18	1.554 (3)
C5—C9	1.428 (3)	C17—H17A	0.9900
C6—N1	1.317 (3)	C17—H17B	0.9900
C6—C7	1.418 (3)	C18—C19	1.505 (3)
C6—H6	0.9500	C18—H18	1.0000
C7—C8	1.367 (3)	C19—C20	1.319 (4)
C7—H7	0.9500	C19—H19	0.9500
C8—C9	1.429 (3)	C20—H20A	0.9500
C8—C11	1.521 (3)	C20—H20B	0.9500
C9—C10	1.419 (3)	N2—H2N	0.9300
			
O4—S1—O4^i^	110.34 (12)	C11—C12—H12	107.4
O4—S1—O5^i^	109.85 (9)	C13—C12—H12	107.4
O4^i^—S1—O5^i^	110.20 (9)	C14—C13—C12	109.47 (17)
O4—S1—O5	110.20 (9)	C14—C13—H13A	109.8
O4^i^—S1—O5	109.85 (9)	C12—C13—H13A	109.8
O5^i^—S1—O5	106.33 (11)	C14—C13—H13B	109.8
C2—O1—C1	116.78 (18)	C12—C13—H13B	109.8
C11—O2—H2	109.5	H13A—C13—H13B	108.2
H3A—O3—H3B	103.5	C15—C14—C18	107.87 (18)
O1—C1—H1A	109.5	C15—C14—C13	108.27 (18)
O1—C1—H1B	109.5	C18—C14—C13	111.57 (18)
H1A—C1—H1B	109.5	C15—C14—H14	109.7
O1—C1—H1C	109.5	C18—C14—H14	109.7
H1A—C1—H1C	109.5	C13—C14—H14	109.7
H1B—C1—H1C	109.5	C14—C15—C16	108.85 (18)
O1—C2—C10	125.74 (19)	C14—C15—H15A	109.9
O1—C2—C3	113.79 (19)	C16—C15—H15A	109.9
C10—C2—C3	120.5 (2)	C14—C15—H15B	109.9
C4—C3—C2	119.8 (2)	C16—C15—H15B	109.9
C4—C3—H3	120.1	H15A—C15—H15B	108.3
C2—C3—H3	120.1	N2—C16—C15	109.17 (17)
C3—C4—C5	121.70 (19)	N2—C16—H16A	109.8
C3—C4—H4	119.2	C15—C16—H16A	109.8
C5—C4—H4	119.2	N2—C16—H16B	109.8
N1—C5—C4	118.38 (19)	C15—C16—H16B	109.8
N1—C5—C9	123.3 (2)	H16A—C16—H16B	108.3
C4—C5—C9	118.4 (2)	N2—C17—C18	109.07 (18)
N1—C6—C7	123.8 (2)	N2—C17—H17A	109.9
N1—C6—H6	118.1	C18—C17—H17A	109.9
C7—C6—H6	118.1	N2—C17—H17B	109.9
C8—C7—C6	119.7 (2)	C18—C17—H17B	109.9
C8—C7—H7	120.2	H17A—C17—H17B	108.3
C6—C7—H7	120.2	C19—C18—C14	113.00 (19)
C7—C8—C9	118.79 (19)	C19—C18—C17	110.20 (19)
C7—C8—C11	120.4 (2)	C14—C18—C17	108.20 (18)
C9—C8—C11	120.7 (2)	C19—C18—H18	108.4
C10—C9—C5	119.3 (2)	C14—C18—H18	108.4
C10—C9—C8	123.71 (19)	C17—C18—H18	108.4
C5—C9—C8	117.0 (2)	C20—C19—C18	124.5 (2)
C2—C10—C9	120.28 (19)	C20—C19—H19	117.8
C2—C10—H10	119.9	C18—C19—H19	117.8
C9—C10—H10	119.9	C19—C20—H20A	120.0
O2—C11—C8	110.29 (18)	C19—C20—H20B	120.0
O2—C11—C12	111.09 (16)	H20A—C20—H20B	120.0
C8—C11—C12	107.66 (17)	C6—N1—C5	117.42 (18)
O2—C11—H11	109.3	C16—N2—C17	108.91 (16)
C8—C11—H11	109.3	C16—N2—C12	115.10 (17)
C12—C11—H11	109.3	C17—N2—C12	107.21 (16)
N2—C12—C11	111.88 (16)	C16—N2—H2N	108.5
N2—C12—C13	108.31 (15)	C17—N2—H2N	108.5
C11—C12—C13	114.11 (19)	C12—N2—H2N	108.5
N2—C12—H12	107.4		
			
C1—O1—C2—C10	0.3 (3)	O2—C11—C12—C13	45.5 (2)
C1—O1—C2—C3	178.89 (18)	C8—C11—C12—C13	−75.4 (2)
O1—C2—C3—C4	−176.14 (19)	N2—C12—C13—C14	−1.2 (3)
C10—C2—C3—C4	2.6 (3)	C11—C12—C13—C14	−126.55 (19)
C2—C3—C4—C5	−1.9 (3)	C12—C13—C14—C15	60.7 (2)
C3—C4—C5—N1	−179.3 (2)	C12—C13—C14—C18	−57.9 (2)
C3—C4—C5—C9	−0.6 (3)	C18—C14—C15—C16	55.2 (2)
N1—C6—C7—C8	1.3 (3)	C13—C14—C15—C16	−65.7 (2)
C6—C7—C8—C9	0.2 (3)	C14—C15—C16—N2	9.5 (2)
C6—C7—C8—C11	−177.01 (19)	C15—C14—C18—C19	171.51 (16)
N1—C5—C9—C10	−179.0 (2)	C13—C14—C18—C19	−69.7 (2)
C4—C5—C9—C10	2.3 (3)	C15—C14—C18—C17	−66.2 (2)
N1—C5—C9—C8	1.6 (3)	C13—C14—C18—C17	52.6 (2)
C4—C5—C9—C8	−177.13 (19)	N2—C17—C18—C19	133.72 (19)
C7—C8—C9—C10	179.07 (19)	N2—C17—C18—C14	9.7 (2)
C11—C8—C9—C10	−3.7 (3)	C14—C18—C19—C20	−123.4 (3)
C7—C8—C9—C5	−1.5 (3)	C17—C18—C19—C20	115.4 (3)
C11—C8—C9—C5	175.70 (18)	C7—C6—N1—C5	−1.3 (3)
O1—C2—C10—C9	177.73 (19)	C4—C5—N1—C6	178.53 (19)
C3—C2—C10—C9	−0.8 (3)	C9—C5—N1—C6	−0.2 (3)
C5—C9—C10—C2	−1.6 (3)	C15—C16—N2—C17	−67.5 (2)
C8—C9—C10—C2	177.8 (2)	C15—C16—N2—C12	52.9 (2)
C7—C8—C11—O2	−16.4 (3)	C18—C17—N2—C16	55.8 (2)
C9—C8—C11—O2	166.46 (18)	C18—C17—N2—C12	−69.36 (19)
C7—C8—C11—C12	105.0 (2)	C11—C12—N2—C16	69.1 (2)
C9—C8—C11—C12	−72.2 (2)	C13—C12—N2—C16	−57.5 (2)
O2—C11—C12—N2	−78.0 (2)	C11—C12—N2—C17	−169.59 (17)
C8—C11—C12—N2	161.20 (17)	C13—C12—N2—C17	63.8 (2)

Symmetry codes: (i) −*x*+1, *y*, −*z*+2.

### Hydrogen-bond geometry (Å, °)

**Table:**

Cg1 and Cg2 are the centroids of the C2–C10 and N1–C5 rings, respectively.

**Table:**

*D*—H···*A*	*D*—H	H···*A*	*D*···*A*	*D*—H···*A*
C11—H11···O4^i^	1.00	2.59	3.584 (3)	171
C16—H16A···O3	0.99	2.36	3.345 (3)	176
C17—H17B···O2^ii^	0.99	2.33	3.174 (3)	143
N2—H2N···O5	0.93	1.77	2.698 (3)	175
O2—H2···O3	0.84	1.87	2.695 (2)	166
O3—H3A···O5^iii^	0.86	1.99	2.765 (3)	149
O3—H3B···O4	0.86	1.97	2.794 (3)	159
C6—H6···Cg2^iv^	0.95	2.67	3.482 (3)	144
C20—H20B···Cg1^v^	0.95	2.85	3.530 (3)	130

Symmetry codes: (i) −*x*+1, *y*, −*z*+2; (ii) *x*, *y*−1, *z*; (iii) *x*, *y*+1, *z*; (iv) −*x*+1/2, *y*+1/2, −*z*+1; (v) *x*−1/2, *y*−1/2, *z*.
